# Enhanced acute absorption of vitamin D3 with a liposomal formulation (Cymbiotika) compared to a standard formulation in healthy adults: a randomized double-blind crossover study

**DOI:** 10.3389/fnut.2026.1858064

**Published:** 2026-10-13

**Authors:** David Briskey, Sandra Rahman, Shilpa Raut, Chervin Jafarieh, Amanda Rao

**Affiliations:** 1 RDC Clinical, Brisbane, QLD, Australia; 2 School of Human Movement and Nutrition Sciences, University of Queensland, Brisbane, QLD, Australia; 3 Cymbiotika LLC, San Diego, CA, United States

**Keywords:** absorption, bioavailability, cholecalciferol, liposomal delivery, vitamin D3

## Abstract

**Background:**

Vitamin D deficiency is increasingly prevalent and linked to impaired health and disease. While vitamin D3 supplementation is effective, variability in absorption can influence bioavailability and clinical responses, with formulation characteristics potentially contributing to this variability.

**Method:**

The aim of this randomized, double-blind, crossover absorption study was to compare the absorption of a liposomal vitamin D3 formulation (Cymbiotika) with that of a conventional non-liposomal vitamin D3 formulation. Healthy adult participants received a single oral dose of both formulations in random order separated by a 2-week washout period. Blood samples were collected over 48 h post-dose and plasma cholecalciferol concentrations quantified. Absorption parameters including area under the concentration-time curve (AUC), maximum plasma concentration (Cmax), and time to peak concentration (Tmax) were calculated following baseline correction.

**Results:**

Twelve participants completed the study (mean age 34.3 ± 9.7 years; BMI 24.8 ± 2.7 kg/m2). The liposomal formulation showed greater systemic absorption compared with the comparator formulation, with a 1.9-fold higher AUC (199 vs. 106 ng·h/mL). Peak concentration was also higher for the liposomal formulation (8.4 vs. 6.0 ng/mL). Circulating cholecalciferol concentrations remained elevated above baseline at 48 h in the liposomal formulation but returned to baseline in the comparator formulation.

**Conclusion:**

These findings suggest that the Cymbiotika formulation may enhance the acute absorption of orally administered vitamin D3. Whether this enhanced acute absorption translates into improved long-term vitamin D status requires investigation in longer-term supplementation studies.

**Clinical trial registration:**

https://doi.org/10.1186/ISRCTN39055102, identifier (ISRCTN39055102).

## 1 Introduction

Vitamin D plays a critical role in calcium and phosphate homeostasis, skeletal integrity, and a wide range of physiological processes including immune function and metabolic regulation (1–3). Despite its importance, vitamin D deficiency remains common globally, affecting a large proportion of the population across both developed and developing regions (1, 3).

Vitamin D3 (cholecalciferol) is the most used form of vitamin D in dietary supplements. After oral ingestion, vitamin D3 is absorbed primarily in the small intestine through mechanisms closely associated with dietary lipid absorption (4, 5). As a highly lipophilic molecule, vitamin D3 requires incorporation into mixed micelles formed by bile salts and dietary lipids prior to uptake by enterocytes (4, 5). Following absorption, vitamin D3 is incorporated into chylomicrons and transported via the lymphatic system into systemic circulation before undergoing tissue distribution and hepatic hydroxylation (6–8). In brief, mixed micelles are colloidal particles formed from bile salts, phospholipids and dietary lipids that solubilise the lipophilic vitamin D3 within the aqueous intestinal lumen, enabling its diffusion to the enterocyte brush border for uptake; chylomicrons are the lipoprotein particles subsequently assembled within enterocytes to package absorbed vitamin D3 together with dietary triglycerides for secretion into the intestinal lymphatics, in contrast to more hydrophilic nutrients that are absorbed directly into the portal blood.

These absorption mechanisms introduce several factors that can influence vitamin D absorption. Dietary fat intake, bile acid secretion, intestinal physiology, and supplement formulation may all influence absorption efficiency (4, 5, 9). Standard vitamin D supplements frequently rely on passive micelle formation during digestion, which can contribute to variability in systemic exposure.

To address these limitations, various formulation strategies have been developed to enhance the absorption of lipophilic nutrients. These approaches include conventional oil-based preparations as well as more specialised delivery systems, such as nanoemulsions, micellar systems, and liposomal formulations (9). Recent advances in nanotechnology have further supported the development of lipid-based nanocarriers, including liposomes, to improve the stability, solubilisation, intestinal uptake and bioavailability of fat-soluble vitamins (10, 11). Liposomes consist of phospholipid bilayer vesicles capable of encapsulating lipophilic compounds and interacting with biological membranes. Recent experimental and clinical evidence suggests that liposomal vitamin D₃ delivery systems may enhance early absorption and systemic exposure compared with conventional non-liposomal formulations (12). By improving solubility and protecting incorporated compounds during gastrointestinal transit, liposomal delivery systems may facilitate intestinal uptake of fat-soluble compounds.

The aim of the present study was therefore to compare the acute absorption profiles of a liposomal vitamin D3 formulation and a conventional non-liposomal vitamin D3 formulation in healthy adults by measuring circulating cholecalciferol concentrations over a 48-h period following administration.

## 2 Methods

### 2.1 Study design

This study was conducted as a randomized, double-blind, crossover absorption trial designed to evaluate the absorption characteristics of two vitamin D3 formulations in healthy adult participants. The study was conducted in January 2026, at RDC Clinical in Brisbane, Australia in accordance with the principles of Good Clinical Practice and national ethical guidelines. This study was registered with ISRCTN (registration number ISRCTN39055102).

Each participant received both the investigational and comparator products in separate study periods and therefore served as their own control. Participants were randomized to receive either the liposomal (Cymbiotika) formulation followed by the comparator formulation or vice versa. The two dosing sessions were separated by a washout period of at least 2-weeks. Randomisation was performed using a computer-generated sequence via https://www.sealedenvelope.com/, with a 1:1 allocation ratio using block randomisation (block size of 4). An independent person not involved in the conduct of the trial generated the randomisation list and maintained allocation concealment in a sealed, locked envelope. Participants, investigators, and laboratory personnel were all blinded to treatment allocation throughout the study. The randomisation code was not revealed until after all sample analysis, including statistical analysis, was complete. No unblinding occurred prior to completion of the analysis.

### 2.2 Participants

Participants eligible for inclusion in this study were healthy adult males and females aged between 18 and 50 years. Individuals were required to have a body mass index (BMI) between 18.5 and 30.0 kg/m^2^ and be in generally good health, as determined by medical history and investigator assessment.

Eligible participants were required to have stable dietary habits and lifestyle patterns for at least 4 weeks prior to enrolment and throughout the study period. Individuals were required to refrain from taking vitamin D supplements or other products known to influence vitamin D metabolism or absorption for at least 2 weeks prior to the first dosing visit and for the duration of the study.

Female participants of childbearing potential were required to use a medically accepted method of contraception for the duration of the study and to confirm that they were not pregnant or breastfeeding at the time of enrolment.

Participants were excluded if they had any medical condition that could interfere with the absorption, metabolism, or distribution of vitamin D. This included, but was not limited to, gastrointestinal disorders such as inflammatory bowel disease, celiac disease, Crohn’s disease, or a history of gastrointestinal surgery affecting nutrient absorption. Individuals with known metabolic, hepatic, renal, cardiovascular, or endocrine disorders were also excluded where these conditions could impact study outcomes or participant safety.

Participants were excluded if they were currently taking medications or supplements known to affect vitamin D metabolism, lipid absorption, or gastrointestinal function, including but not limited to vitamin D supplements, high-dose multivitamins, bile acid sequestrants, or lipid-lowering agents, within 2 weeks prior to study commencement. Individuals with known sensitivity to any components of the study products were also excluded.

Additional exclusion criteria included participation in another clinical trial within the previous 30 days, significant changes in diet or lifestyle within 4 weeks prior to enrolment, or any other condition that, in the opinion of the investigator, would make participation unsuitable or compromise the integrity of the study data.

All participants provided written informed consent prior to participation.

### 2.3 Study interventions

Two vitamin D3 formulations were evaluated in this study. Both formulations contained identical doses of vitamin D3 (4,000 IU) and vitamin K2 (620 μg). The Cymbiotika liposomal D3 investigational product consisted of a liposomal vitamin D3 formulation incorporating phospholipid carriers designed to enhance solubilisation and intestinal uptake. The Cymbiotika product was administered as a phospholipid-based gel formulation with an aqueous vehicle consisting predominantly of water (approximately 70–80%) and glycerin. During manufacture, vitamin D3 and vitamin K2 were dispersed with sunflower lecithin-derived phosphatidylcholine and incorporated into the water-glycerin vehicle, followed by homogenization to form the final liposomal formulation. The Cymbiotika gel additionally contained coenzyme Q10 (40 mg per sachet) as a co-formulated ingredient, together with other inactive excipients (e.g., glycerin, tocopherol, natural flavourings) required for the gel delivery format. The Cymbiotika formulation was supplied in individual pre-measured sachets, with each sachet containing a single fixed dose as predetermined by the manufacturer. Participants consumed the entire contents of one sachet at each dosing occasion, thereby providing 4,000 IU of vitamin D3, 620 μg of vitamin K2, and 40 mg of coenzyme Q10 per administered dose.

The tablet comparator was prospectively selected as it represents a common commercially available form of oral vitamin D3 that is a non-liposomal formulation and provided matched doses of vitamin D3 and vitamin K2. The comparator was therefore intended to provide a pragmatic reference against which to evaluate the complete investigational formulation, rather than to provide a dosage-form-matched control. The comparator tablet did not contain coenzyme Q10 or a phospholipid/liposomal carrier. The comparator tablet also contained: microcrystalline cellulose, stearic acid, croscarmellose sodium, magnesium stearate, silicon dioxide, hydroxypropyl methylcellulose, and polyethylene glycol. Both the comparator (tablet form) and Cymbiotika (gel form) products contained cholecalciferol (vitamin D3).

Participants, investigators, laboratory personnel, and the statistician were blinded to treatment allocation. As the investigational and comparator products differed in dosage form (liquid gel and tablet), an independent, unblinded staff member prepared and administered study treatments according to the randomisation schedule. Treatment allocation remained concealed until database lock. The comparator was a commercially available, non-liposomal cholecalciferol supplement purchased from a USA pharmacy. The manufacturer is not disclosed due to proprietary considerations; however, the comparator contained the same dose of cholecalciferol (4,000 IU) and vitamin K2 (620 μg) as the investigational product. Beyond matching the active ingredient doses, the two products could not be matched for all formulation characteristics, as the study was designed to compare commercially available products in their marketed forms. Consequently, the formulations differed in several characteristics, including dosage form (gel versus tablet), excipient composition, phospholipid carrier, and the presence of co-formulated coenzyme Q10. The potential influence of dosage form was therefore not experimentally isolated or negated. Rather, the study was designed to compare the overall acute absorption performance of the complete formulations, and any observed differences may reflect the combined effects of dosage form, carrier system, and other compositional differences between the products.

Participants attended the clinical research facility following an overnight fast (>10-h). After baseline blood sampling, participants consumed the assigned study product with approximately 250 mL of water. A standardised breakfast meal (approximately 30% fat, 40% carbohydrate and 30% protein) was provided to participants approximately 30 min after dosing. The products were administered before breakfast to minimise the influence of concurrent food, particularly dietary fat, during the initial post-dose absorption period. Although dietary fat may influence vitamin D3 absorption, the magnitude and direction of any food effect on the relative absorption of the two formulations were not known and were not an objective of the present study. Concurrent administration with food could therefore have introduced an additional formulation-by-food interaction into the comparison. Separating product administration from breakfast was intended to reduce this potential source of variability and allow the acute absorption profiles to more directly reflect differences between the complete formulations under standardised dosing conditions. Breakfast was subsequently provided to reflect a realistic morning feeding pattern and was identical across treatment periods. Lunch and snacks provided during the clinic visit on Day 1 were similarly standardised and nutritionally balanced (approximately 50% carbohydrate, 20% protein, and 30% fat). All meals consumed during the clinic visit were identical across participants. Beyond the clinic visit, participants were advised to maintain their habitual dietary patterns but were not restricted in their food choices for the 24- and 48-h follow-up periods.

### 2.4 Blood sampling

Venous blood samples were collected via an indwelling cannula placed in the antecubital vein. Collected blood was immediately transferred to EDTA vacutainers and stored at 4 °C prior to centrifugation. Baseline samples were collected prior to ingestion of the study product. Subsequent blood samples were obtained at 0.5, 1, 2, 4, 6, 8, and 10 h following supplementation. Additional samples were obtained at 24 and 48 h post-dose.

Blood samples were centrifuged at 1,600 × g for 10 min at 4 °C to separate plasma. Plasma samples were aliquoted and stored at −80 °C until analysis.

### 2.5 Analytical methods

Plasma concentrations of vitamin D3 (cholecalciferol) were quantified using a commercially available competitive enzyme-linked immunosorbent assay (ELISA) specific for cholecalciferol (Invitrogen Vitamin D3 Competitive ELISA Kit, Cat. No. EEL151; ThermoFisher Scientific, Victoria, Australia). Samples were analysed in duplicate according to the manufacturer’s instructions. The kit has a reported sensitivity of 0.94 ng/mL and a working range of 1.56–100 ng/mL, employing a competitive inhibition format. Circulating cholecalciferol was selected as the primary analyte as it reflects acute systemic exposure following intestinal absorption, whereas 25-hydroxyvitamin D reflects downstream hepatic metabolism and is therefore not suitable for assessing acute absorption following a single dose.

### 2.6 Absorption and statistical analysis

Because vitamin D3 is an endogenous compound present in circulating plasma prior to dosing, baseline correction was applied to account for pre-existing concentrations. For each participant, the pre-dose concentration was subtracted from all post-dose concentrations to isolate the absorption attributable to the dose. Negative values resulting from baseline correction were set to zero. This approach is consistent with standard methodology for pharmacokinetic studies involving endogenous analytes. Negative post-correction values reflect random biological fluctuation around the pre-dose baseline rather than true negative absorption and setting these to zero prevents artefactual reduction of the AUC estimate. While this truncation may introduce a degree of upward bias in AUC estimates, the approach was applied consistently across both treatment periods and both formulations; any bias therefore affects both arms equally and would not be expected to systematically favour one treatment. This methodology was pre-specified in the study protocol and no post-hoc adjustments were made.

The primary absorption parameter was the area under the plasma concentration-time curve from time zero to 48 h (AUC_0–48h_). AUC values were calculated using the linear trapezoidal rule. Secondary absorption parameters included maximum observed plasma concentration (Cmax) and time to maximum plasma concentration (Tmax). Absorption parameters were calculated individually for each participant and treatment period. A one-tailed paired t-test was pre-selected for this study on the basis of the pre-specified directional hypothesis that the liposomal investigational formulation was intended to increase systemic exposure relative to the conventional comparator formulation, giving a directional *a priori* hypothesis, that is, a hypothesis specifying in advance the expected direction of the treatment difference, namely, that the AUC of the liposomal formulation (denoted A) would exceed that of the comparator formulation (denoted B).

The sample size was determined *a priori* based on published pharmacokinetic data describing the variability of vitamin D₃ absorption following single-dose supplementation. As no directly comparable data were available for the present study design or primary endpoint (baseline-corrected AUC_0–48h_), the closest available published pharmacokinetic estimates were used to generate the sample size calculation. Assuming a two-period crossover design, a within-subject coefficient of variation of approximately 12, 80% statistical power, and a one-sided significance level of 0.05, a minimum of 12 participants completing both study periods was estimated to be sufficient to detect a 15% difference in AUC between formulations. To allow for potential withdrawals, 16 participants were enrolled, with 12 completing both study periods and included in the primary analysis.

Continuous variables were summarised as mean ± standard deviation. Prior to statistical analysis, normality of AUC and Cmax distributions was assessed using histograms and quantile-quantile (QQ) plots. Differences in AUC and Cmax between formulations were evaluated using a paired one-sided t-test. Tmax was compared using a Wilcoxon signed-rank test. As a sensitivity analysis, a linear mixed-effects model including treatment, period and sequence as fixed effects and participant was included as a random effect. The mixed-model results were consistent with the primary paired analyses. No significant treatment, sequence, period, or carryover effects were detected. Analyses were performed using SPSS (Version 31; IBM Corp., Armonk, NY, USA) and GraphPad Prism (Version 11, San Diego, CA, USA). Statistical significance was defined as *p* < 0.05.

## 3 Results

### 3.1 Participant characteristics

Twelve participants completed both study periods (Figure 1) with participant demographics summarized in Table 1.

**Figure 1:**
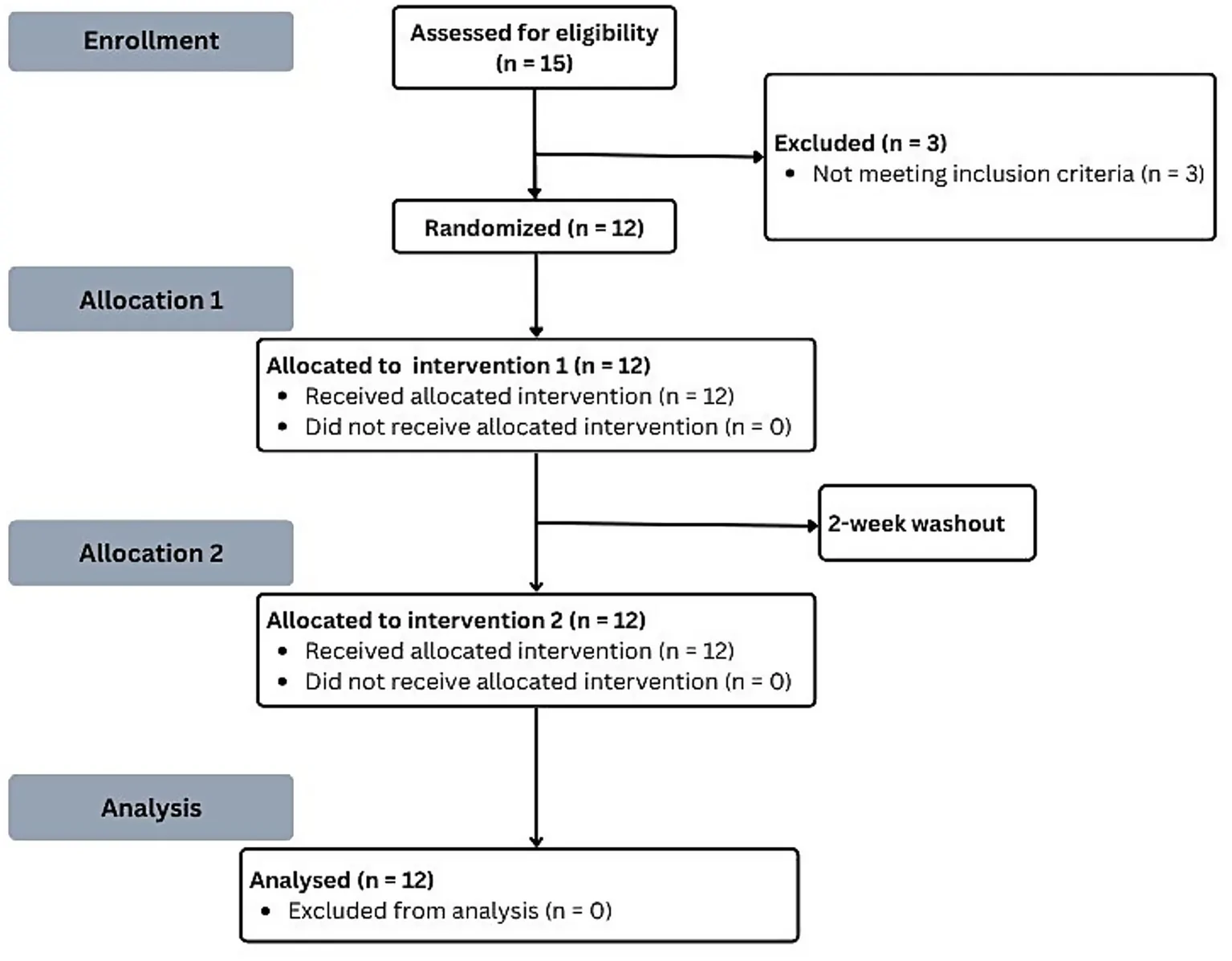
CONSORT flow diagram of participant progress through the trial. Values are presented as mean ± standard deviation or *n* (%).

**Table 1:** Participant demographics and baseline characteristics.

Characteristic	Value (*N* = 12)
Age (years)	34.25 ± 9.69 (range: 18–49)
Gender (n)	Female: 7 (58.3%), Male: 5 (41.7%)
BMI (kg/m^2^)	24.75 ± 2.70 (range: 20.85–28.47)
Height (m)	1.71 ± 0.10
Weight (kg)	72.66 ± 14.45
Systolic BP (mmHg)	116.58 ± 11.65
Diastolic BP (mmHg)	73.67 ± 7.85
Pulse (bpm)	66.08 ± 23.17
Ethnicity (*n*)	SE Asian: 2 (16.7%), Caucasian: 4 (33.3%), E Asian: 1 (8.3%), Hispanic: 3 (25.0%), S Asian: 1 (8.3%), European: 1 (8.3%)

Baseline plasma cholecalciferol concentrations were well matched across both doses. The liposomal formulation had a baseline of 76.7 ± 42.0 ng/mL (range: 30.8–132.9 ng/mL, *n =* 12). The comparator had a baseline of 77.9 ± 50.7 ng/mL (range: 29.6–174.4 ng/mL, *n* = 12). The wide range in baseline cholecalciferol concentrations reflects typical inter-individual variation in a healthy adult population. The crossover design, in which each participant acted as their own control, mitigates the potential confounding effect of baseline concentration. Exploratory correlation analysis between individual baseline concentrations and AUC or Cmax values did not reveal a clear directional relationship.

### 3.2 Absorption outcomes

No significant sequence, period, or carryover effects were identified in the mixed-effects analysis (all *p* > 0.05).

The liposomal formulation demonstrated a significant increase in AUC of approximately 1.9-fold compared with the comparator formulation (Table 2). Circulating cholecalciferol concentrations remained elevated above baseline at 48 h following the liposomal formulation but had returned to baseline in the comparator formulation (Figure 2). Individual participant AUC_0–48h_ values showed considerable inter-individual variability, consistent with known biological variation in fat-soluble vitamin absorption, which can be influenced by factors such as inter-individual differences in bile acid secretion, gastric emptying rate, intestinal transit time, and adiposity; this variability is a recognised feature of vitamin D pharmacokinetic studies and was mitigated, though not eliminated, by the crossover design in which each participant served as their own control. For the liposomal formulation, individual AUC values ranged from 0 to 431.6 ng·h/mL; for the comparator formulation, values ranged from 0 to 328.2 ng·h/mL. The mean paired treatment difference (liposomal − comparator) was 92.7 ng·h/mL (95% CI: −13.2 to 198.7 ng·h/mL; paired t-test: t = 1.926, df = 11, *p* = 0.040 one-tailed; Cohen’s *d* = 0.56). The Cmax comparison also reached statistical significance (*p* = 0.033 one-tailed). No adverse events related to either product were reported.

**Table 2:** Absorption parameters following administration of Cymbiotika and comparator vitamin D3 formulations.

Parameter	Liposomal (*n* = 12)	Comparator (*n* = 12)
AUC_0–48h_ (ng·h/mL)	199 ± 149^a^	106 ± 84
Fold difference^b^	1.9	N/A
95% CI	−13.2 to 198.7	
Cmax (ng/mL)	8.4 ± 3.2	6.0 ± 2.5
Tmax (h)	1.0	0.5

**Figure 2:**
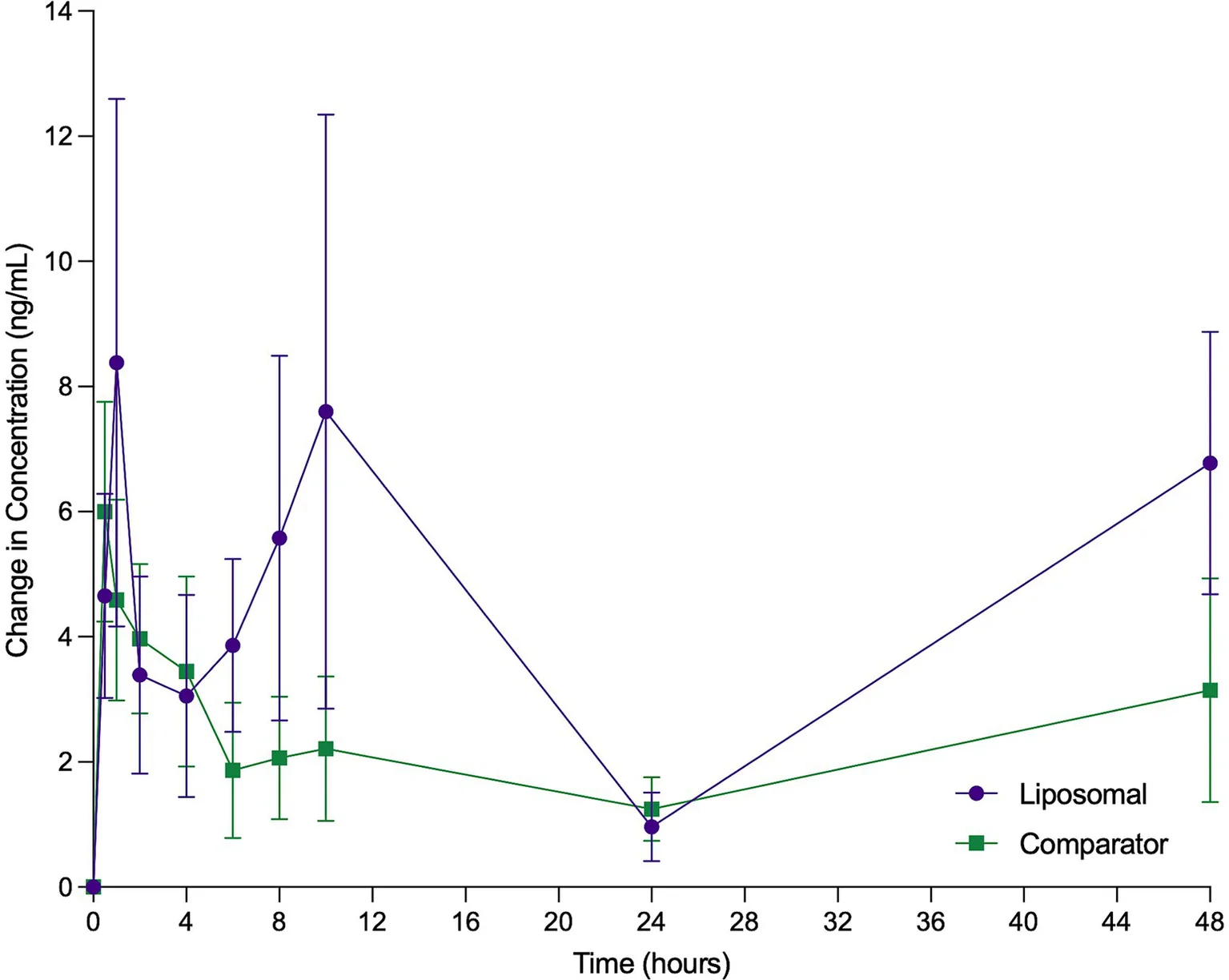
Change from baseline comparative absorption of vitamin D between formulations. Total AUC was significantly greater in the liposomal formulation compared to the comparator formulation (*p* < 0.05). ^a^statistically significant difference between formulations (*p* < 0.05); ^b^Compared to comparator; statistical significance between formulations = *p* < 0.05. paired *t*-test: *t* = 1.926, df = 11, *p* = 0.040 one-tailed; Cohen’s *d* = 0.56. Values are presented as mean ± standard deviation.

## 4 Discussion

The present study assessed the acute absorption of Cymbiotika liposomal vitamin D3 compared with a standard, tablet-based vitamin D3 supplement (comparator) in healthy adults. The results demonstrate that the Cymbiotika formulation produced greater acute absorption of cholecalciferol, as reflected by higher AUC and Cmax values.

Several studies have demonstrated that formulation characteristics may significantly influence the intestinal absorption of vitamin D3, independent of dose. Differences in the physical presentation of vitamin D3, including oil-based preparations, emulsions, micellar systems and liposomal formulations, influence solubilisation, incorporation into mixed micelles, and subsequent uptake by enterocytes, resulting in differences in systemic exposure (9–12). The comparator tablet contained croscarmellose sodium, a commonly used superdisintegrant that promotes rapid tablet disintegration and may facilitate release of the active ingredient following ingestion. Together with the early Tmax of 0.5 h, this indicates that the lower AUC and Cmax of the comparator were unlikely to be explained solely by a delayed physical disintegration of the tablet. Rather, the differences in systemic exposure may reflect other characteristics of the complete formulations that influence vitamin D3 solubilisation and intestinal uptake. Separately, taking vitamin D with a meal containing fat has been associated with improved absorption through stimulation of normal digestive lipid processing (13, 14). In the present study, participants received the study products in the fasted state, followed by a standardised breakfast approximately 30 min after dosing. This approach was selected to minimise the influence of concurrent food intake during the initial absorption period. Although dietary fat can influence vitamin D3 absorption, it is not known whether concurrent food intake would have affected the magnitude of absorption of the two formulations equally or differently. The study was not designed to evaluate such formulation-by-food interactions. Accordingly, separating dosing from breakfast reduced food intake as a potential compounding factor and allowed the observed differences to be interpreted primarily as differences between the complete formulations under the standardised conditions employed in this study.

Another important consideration in vitamin D absorption research relates to the biomarker used to assess absorption. Most clinical trials evaluating vitamin D supplementation measure circulating 25-hydroxyvitamin D, which is widely accepted as the best indicator of long-term vitamin D status (15–17). However, 25(OH)D reflects hepatic metabolism and has a half-life of approximately 2 to 3 weeks (18–20). Although 25(OH)D is widely accepted as the primary biomarker of vitamin D status, it is not optimal for evaluating acute absorption following single-dose supplementation because it reflects hepatic conversion and a longer biological half-life rather than immediate intestinal uptake (18–20). Therefore, measurement of 25(OH)D is not ideal for evaluating acute absorption responses following single-dose supplementation.

For a single-dose absorption study focused on early absorption, circulating cholecalciferol is likely the more appropriate analyte. Circulating cholecalciferol concentrations rise rapidly following oral vitamin D3 ingestion and reflect the immediate intestinal absorption phase (19, 20). Plasma cholecalciferol typically reaches peak concentrations within several hours before declining as the compound is distributed to tissues and metabolized. Measurement of cholecalciferol therefore provides a sensitive marker for assessing intestinal uptake during short-term absorption studies.

Previous absorption studies have used circulating vitamin D3 concentrations or closely related metabolic responses to examine absorption following supplementation. Ilahi et al. demonstrated that circulating vitamin D concentrations rise sharply within hours of a single large oral dose of cholecalciferol before declining over the following days, reflecting a rapid absorption and redistribution phase rather than a sustained plateau (20, 21). Armas et al. and Trang et al. reported that vitamin D3 produced greater and/or more sustained increases in circulating 25-hydroxyvitamin D than equivalent vitamin D2 supplementation, demonstrating that the two forms differ in their resulting systemic exposure and persistence in humans (22, 23). A later systematic review and meta-analysis also supported the greater efficacy of vitamin D3 relative to vitamin D2 for improving vitamin D-related exposure and status measures (24).

The approximately 1.9-fold higher AUC observed with the Cymbiotika liposomal formulation indicates greater overall systemic exposure to cholecalciferol following the single dose of vitamin D3. Although peak concentrations occurred early in both formulations, AUC_0-48h_ captures the total circulating exposure over the complete sampling period rather than the rate at which the peak concentration was reached. The comparator reached its observed maximum concentration earlier than the Cymbiotika formulation (median Tmax 0.5 versus 1.0 h, respectively), indicating that the present findings should not be interpreted as demonstrating faster absorption with the Cymbiotika formulation. Rather, the higher Cmax and AUC_0-48h_ indicate greater systemic exposure under the conditions of this study. This is biologically plausible given prior work showing that vitamin D absorption is enhanced by favourable lipid environments and that formulation effects can materially alter absorption (9, 13, 14). Although the present study did not directly compare liposomal delivery with micellar or nanoemulsion systems, the findings are directionally consistent with the broader concept that lipid-based delivery approaches may improve absorption of fat-soluble compounds. More recent work has shown that rationally designed liposomal and nano-sized vitamin D delivery systems improve physicochemical stability, mucus penetration and intestinal uptake, providing mechanistic support for the improved absorption observed in the present study (10–12).

An additional observation was the non-monotonic concentration-time profile of the Cymbiotika formulation, with decreases at intermediate sampling points followed by subsequent increases. These fluctuations should be interpreted cautiously and cannot be considered a desirable or intended characteristic of the formulation based on the present study. Following intestinal absorption, circulating cholecalciferol undergoes dynamic distribution between plasma, lipoproteins and lipid-rich tissues, together with ongoing metabolism and clearance. Accordingly, secondary fluctuations in circulating concentrations could potentially reflect redistribution and other physiological processes rather than continued intestinal absorption. However, the present study was not designed to distinguish among these mechanisms, and the relatively small sample size and discrete sampling intervals late in the study also limit interpretation of individual changes in the mean concentration-time profile. The apparent secondary increases should therefore not be attributed specifically to redistribution from adipose tissue without further mechanistic investigation.

The broader physiological and clinical interpretation of these findings are yet to be determined. Acute enhancement of cholecalciferol exposure does not automatically equate to superior long-term vitamin D status or clinical outcomes (16, 25). Vitamin D physiology is complex, involving transport, hydroxylation, tissue distribution, feedback regulation, and downstream functional responses (7, 24, 26). For this reason, future studies should assess whether the improved acute absorption profile observed here translates into greater or more sustained increases in plasma 25-hydroxyvitamin D during chronic supplementation. Several limitations of the present study should be acknowledged. The sample size of 12 completers is modest, and while the crossover design optimises statistical power, confidence intervals for the treatment difference are wide, reflecting substantial biological variability in vitamin D absorption. The standard errors around the mean concentration-time profile (Figure 2) were similarly wide at most timepoints, again reflecting this inter-individual variability rather than measurement imprecision. Despite this variability, the paired, within-subject crossover design was able to detect a statistically significant treatment difference, and the findings should be interpreted as hypothesis-generating for larger, confirmatory studies rather than as definitive population estimates. Dietary intake outside the clinic visit was not fully controlled; while all clinic meals were standardised, participants consumed ad libitum at home. As a single-dose acute absorption study, no conclusions regarding long-term vitamin D status can be drawn. Liposomal formulations vary in phospholipid composition, vesicle size, and manufacturing; findings with this specific product may not generalise to other liposomal vitamin D supplements. The liposomal formulation additionally contained coenzyme Q10 (40 mg per dose) as a co-formulated ingredient, which was not present in the comparator. The present study was not designed to isolate the contribution of individual formulation characteristics, including the difference in dosage form (gel versus tablet), the aqueous-based gel vehicle, liposomal/phospholipid carrier system, excipient composition, or the presence of co-formulated coenzyme Q10. Accordingly, differences in systemic exposure may reflect the combined influence of these formulation characteristics, and the observed differences in absorption cannot be attributed solely to the liposomal component. Therefore, the study outcomes should be interpreted as reflecting the overall performance of the complete formulations.

## 5 Conclusion

This comparative absorption study demonstrates enhanced acute cholecalciferol absorption with a liposomal delivery technology (Cymbiotika) compared to a standard, tablet-based non-liposomal formulation in healthy adults. The Cymbiotika liposomal formulation achieved an approximately 1.9-fold higher AUC_0–48h_ (199 ng·h/mL) compared to the comparator (106 ng·h/mL), reflecting greater intestinal uptake following a single dose. As this was an acute absorption study measuring circulating cholecalciferol, whether this enhanced absorption profile translates into improved long-term vitamin D status (as assessed by 25-hydroxyvitamin D) would require investigation in future longitudinal studies.

## Data Availability

The raw data supporting the conclusions of this article will be made available by the authors, without undue reservation.
